# Bringing *Lived Lives *to Swift’s Asylum: a psychiatric hospital perspective

**DOI:** 10.12688/wellcomeopenres.15588.1

**Published:** 2021-04-19

**Authors:** Kevin M. Malone, Eimear Cleary, Cecily C. Kelleher, Janis Jefferies, Abbie Lane, James V. Lucey, Seamus McGuiness

**Affiliations:** 1 Department of Psychiatry, University College Dublin, Dublin, Co. Dublin, Ireland; 2 Goldsmith College London, London, UK; 3 Department of Psychiatry, St. Patrick's University Hospital, Dublin, Co. Dublin, Ireland; 4 5GMIT Centre for Creative Arts & Media, Galway Mayo Institute of Technology, Galway, Ireland

**Keywords:** Stigma, Mental Illness, Psychiatric Hospitalization, Suicidal Ideation, Psychoeducation

## Abstract

**Background:** Few “interventions” around suicide and stigma have reached into psychiatric institutions. *Lived Lives* is a science-arts approach to addressing suicide and stigma, informed by a psychobiographical and visual arts autopsy. The resulting artworks and mediated exhibition ( *Lived Lives*), with artist, scientist and the *Lived Lives* families, co-curated by communities, has facilitated dialogue, response and public action around stigma-reduction, consistent with a community intervention. Recent evidence from *Lived Lives* moved us to consider how it may situate within a psychiatric institution, where stigma is chronically apparent.

**Methods:** *Lived Lives* manifested in St. Patrick’s University Hospital (Ireland’s oldest and largest psychiatric hospital) in November 2017.  The mediated exhibition was open to the public for 4 days. Audiences included service users, policy makers, health professionals, senior hospital administrators and members of the public. Opinions and feelings were collected. The event was documented. Bereavement support was available. A Clinician and an artist provided independent evaluation.

**Results:**  86 participants engaged with the exhibition. 62% of participants were suicide-bereaved; 46% had experienced a mental health difficulty, and 35% had been suicidal in the past. 91% thought *Lived Lives* could be of benefit in the aftermath of a suicide death. Half of participants thought *Lived Lives* could help reduce suicidal feelings, whereas 88% thought it could benefit those with Mental Health difficulties. The emotional response was of a visceral nature, including fear, anger, sadness, disgust and anxiety. Bereavement support was occasionally called upon in a supportive capacity.

**Conclusions:** *Lived Lives* sits comfortably in discomfort, unafraid to call out the home-truths about stigma and its pervasive and pernicious impact, and with restoring identity at its core. *Lived Lives* can operate within a psychiatric hospital, as well as in community. The challenge is to move it forward for greater exposure and impacts in at-risk communities.

## Introduction and context

### Depression and suicide in Ireland

Over the past decade (2005 – 2016), there has been an average of 590 suicide and open verdict deaths per year in Ireland (504 suicide deaths, 86 open verdict according to the CSO) ( Central Statistics Office, 2019). International research estimates that between 30% and 60% of suicide deaths suffer from a major depressive disorder (MDD) ( Conwell *et al.*, 1996; Foster *et al.*, 1997; Harwood *et al.*, 2001; Henriksson *et al.*, 1993). Assuming a 60% suicide rate ( Hasin *et al.*, 2018), in excess of 300 suicide deaths in Ireland annually suffer from MDD. (approximately 60 females, 242 males). It has been estimated that every suicide death impacts 71 members of society ( Mallon & Galway, 2015). Therefore, each year, in excess of 20,000 living individuals in Ireland are impacted by an MDD-related suicide death. This impact (or toll) is cumulative, such that over a decade, in excess of 200,000 living individuals in Ireland carry this loss. On the other hand, the vast majority of those suffering from MDD each year do not die by suicide. For every one suicide death each year with MDD in Ireland, there are anywhere between 800 and 1,100 people living with MDD (based on a 12 month prevalence of MDD in 18–64 year olds of 8–12% – 65% female, 35% male). Effective in-patient and out-patient treatment for MDD significantly lowers the risk of suicide ( Brodsky *et al.*, 2018; Möller & Henkel, 2005). However, between 40% and 60% of those who die by suicide do so at the 1 ^st^ attempt ( Bostwick *et al.*, 2016), and therefore never benefit from all the post-attempter intervention (in-patient or out-patient) and treatment protocols (and statutory and voluntary investments). Stigma of mental illness negatively impacts recovery ( Corrigan & Miller 2004; Henderson *et al.*, 2014). There is very little research on projects which challenge the stigma around mental illness and suicide ( Carpiniello & Pinna, 2017; Rüsch *et al.*, 2005).

Stigma has been succinctly defined as “a mark or sign of disgrace, usually eliciting negative attitudes to its bearer” ( Thornicroft *et al.*, 2007). It is a pervasive feature of mental illness which can lead to negative discrimination. The three stigma-fuelling elements relate to knowledge / (ignorance), attitudes / (prejudice) and behaviour / (discrimination) ( Thornicroft *et al.*, 2007). There are many shades of stigma including labelling and stereotyping ( Lai *et al.*, 2001). Patients with mental illness are frequently labelled “frightening”, “unpredictable”, “dangerous” and “strange”. ( Lundberg *et al.*, 2007). Patients with depression are often labelled “emotionally weak”, “inefficient”, “unproductive” and “lazy” ( Lai *et al.*, 2001). Patients who experience or express suicidal tendencies are labelled “bad”, “unbalanced” and “selfish” ( Carpiniello & Pinna, 2017). Families of patients with mental illness, suicidal tendencies and who have been psychiatrically hospitalized are “guilty” by association, and are euphemistically referred to as experiencing “courtesy stigma” ( Phelan & Bromet, 1998). Families who have lost a loved one to suicide from such illness also experience stigma and its attendant silence, secrecy and shame ( Corrigan & Miller, 2004). For those patients who have been under in-patient care, such is the level of stigma that patients can experience being socially ostracised for years after a single hospital admission, thus bearing the stigma of mental illness and the stigma of having been institutionalised for their illness ( Link *et al.*, 1987). The immediate aftermath of psychiatric hospital discharge can be especially challenging for patients coming to terms with their illness, and it constitutes a significant period of risk for suicide ( Mann *et al.*, 2005), where stigma and fears of relapse are frequently factors, sharply contrasting with the care and attention (and investment) afforded those recently discharged from medical / surgical care ( Moses, 2014). Whilst much-needed research is on-going related to understanding and treating major psychiatric disorders, there is much less focus on processes associated with stigma and its impacts in terms of how it is still endemic in (and following) institutional care, and how it may undermine recovery ( Henderson *et al.*, 2014).

***Lived Lives project.*** There are currently very few “interventions” around suicide and stigma with evaluation as an integral component, and fewer still that have reached into psychiatric institutions. According to the Wellcome Trust, Science - Arts collaborations are “especially effective for complex health / society challenges to generate new knowledge and understanding” ( Malone *et al.*, 2017). *Lived Lives* is a mixed methods science-arts approach to addressing suicide and stigma. It has been informed by a combined psychobiographical autopsy and visual arts autopsy for studying suicide and its aftermath. *The Suicide in Ireland Survey* (2003–2008) documented the lives of 104 suicide victims from around Ireland as narrated by their families ( Malone, 2013), and “Lived Lives”, a parallel visual arts autopsy was conducted, in which these suicide-bereaved families donated images and objects related to the deceased to the project, creating an interdisciplinary research platform between Art and Science, which speaks a universal language ( Malone *et al.*, 2017). The resulting artworks and mediated exhibition ( *Lived Lives*), with artist, scientist and the *Lived Lives* families, co-curated by communities has manifested publicly both nationally and internationally over the past decade, engaging bereaved at-risk communities, the public (including some “at-risk” publics – eg Travellers) and suicide prevention policy-makers, and has facilitated dialogue, response and public action around stigma reduction and health promotion ( Malone *et al.*, 2017). Counselling support is provided as per the original ethics protocol. *Lived Lives* is embedded in the Donegal Suicide Prevention implementation plan as part of Connecting for Life – Ireland’s National Suicide Prevention Plan (2015–2020). Independent evaluations have indicated that the project contains components consistent with a community intervention.

***Lived Lives Paper I*.** *Lived Lives* recently manifested in a rural Irish community which included participants who had previous suicidal thoughts (See *Lived Lives* Paper I Cleary *et al.*, 2021 https://doi.org/10.12688/wellcomeopenres.15613.1). Findings from this project indicated that *Lived Lives* creates a “safe-space” for catharsis, dialogue, disclosure and discussion around feelings, and where there is no guilt or secrecy about experiencing or disclosing past suicidal thoughts or acts. With this in mind, we were moved to consider how *Lived Lives* may respond and be responded to within the context of manifesting in a psychiatric institution (St. Patrick’s University Hospital (SPUH), where stigma is chronically apparent both within and without *(Lived Lives* Paper II). A brief description of *Lived Lives* artworks follows. For a more comprehensive description of artworks, see *Lived Lives* Paper I ( Cleary *et al.*, 2021 https://doi.org/10.12688/wellcomeopenres.15613.1) and *Lived Lives: From Tory Island to Swift’s Asylum* E-publication ( McGuinness & Malone, 2019: https://bit.ly/35hUjU6)

**Figure 1.**
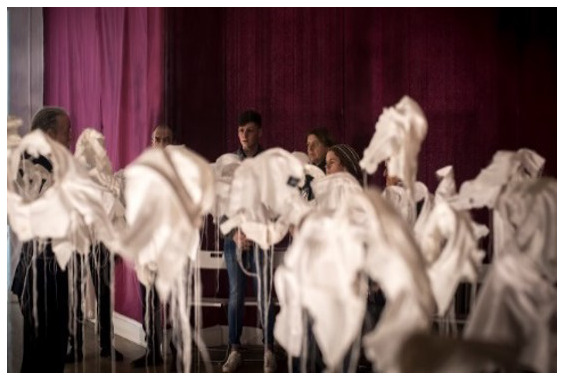
*21g* installed in Group Therapy Room, St. Patrick’s University Hospital.

**Figure 2.**
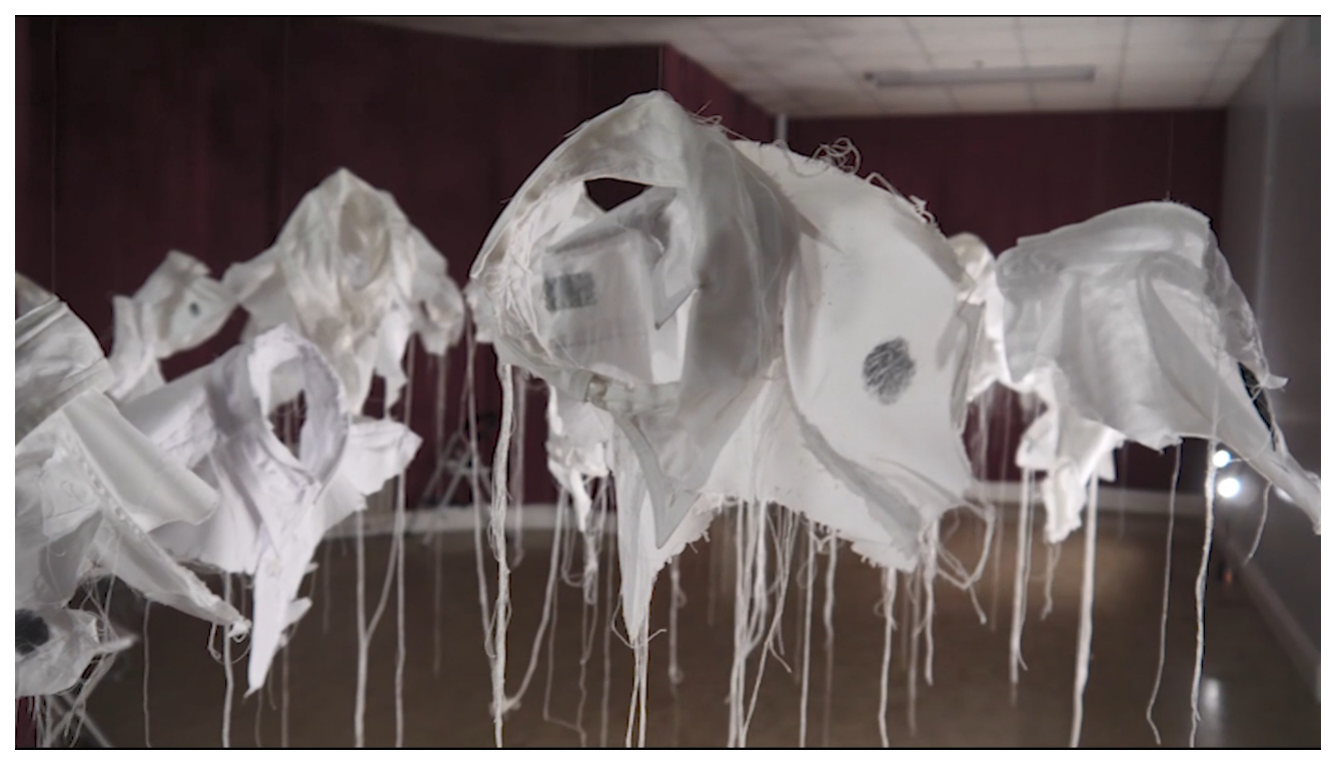
“ *21g -Making Stigma Visible*” installed in Group Therapy Room, St. Patrick’s University: **a**) *Lived Lives* participant marking a shirt fragment upon *21g*; **b**) *21g* shirt fragments marked with ink by *Lived Lives* participants, as metaphor for stigma.

***Lived Lives: Art Installations in St. Patrick’s University Hospital*.** *21 g* ( Figure 1. *21g* installed in Group Therapy Room, St. Patrick’s University Hospital). McGuinness originally created *21g* in 2003 as a visual representation of young male suicide in Ireland in that year. *21 g* consists of in excess of 92 sculpted fragments of cloth (white shirt collars) installed in an anonymous grouping at differing heights, suspended from invisible threads, one for each young male death that year, and each one weighing exactly *21 g*, the mythical weight of the human soul. It is a social probe which asks questions about our knowledge (or the lack of it) about suicide. (See Paper I for images, Cleary *et al.*, 2021). A new art-work – *21g Making Stigma Visible* in SPUH involved participants placing their inked finger on one of the *Lived Lives* shirt fragments as a tactile and tangible metaphor for stigma ( Figure 2. “ *21g -Making Stigma Visible*” installed in Group Therapy Room, St. Patrick’s University: a) *Lived Lives* participant marking a shirt fragment upon *21g*; b) *21g* shirt fragments marked with ink as metaphor for stigma)

**Figure 3.**
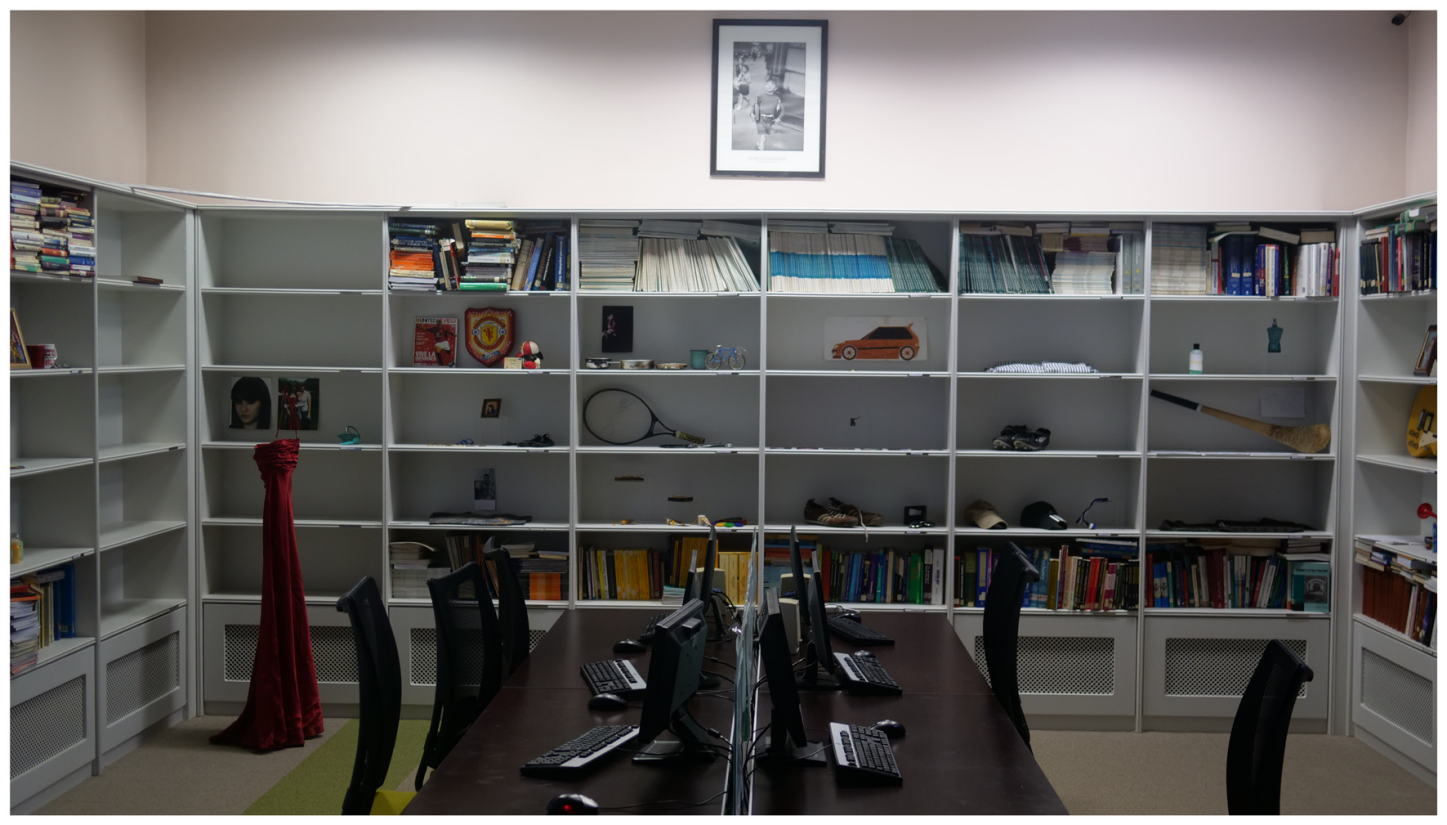
*Archive Rooms,* installed in the Medical Library, St. Patrick’s University Hospital.

*Archive Rooms* consists of artworks constructed by the artist with clothing, writing and other personal objects belonging to the deceased, donated by the participating families, which were originally exhibited as a series of rooms with each loved lost one being represented by their individual belongings. It was installed in the Medical Library for this event ( Figure 3. *Archive Rooms,* installed in the Medical Library, St. Patrick’s University Hospital).

**Figure 4.**
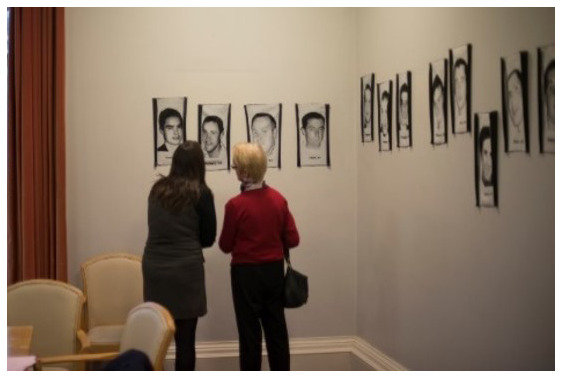
Participants engaging with *The Lost Portraits Gallery* installed in the Conference Room, St. Patrick’s University Hospital.

*The Lost Portrait Gallery* consists of 39 jacquard (tapestry) portraits of young suicide deceased from the *Lived Lives* families. These jacquards are woven, worked from donations of snapshots and memorial cards given by the families and friends to the *Lived Lives* Archive. They are a photographic representation in cloth of the deceased. Each jacquard measures 36cm x 22cm and is installed at exactly the height of the deceased individual, with the first name and age of the deceased woven below the portrait. The portraits are installed in a round room chronologically according to age (with a few interruptions – see *Lived Lives* Paper I for images). For SPUH *Lived Lives* exhibit, the *Lost Portrait* tapestries were installed in the Hospital Conference Room. ( Figure 4. Participants engaging with the *Lost Portrait Gallery* installed in the Conference Room, St. Patrick’s University Hospital).

*Informed Consent* (See Paper 1 for Image) consists of 106 signatures from families giving signed informed consent to participate in the *Lived Lives* Project. (This was not installed in SPUH due to shortage of space in the venue – see Paper I, ( Cleary *et al.*, 2021).

Following the creation of these artworks, and following the *Lived Lives* families private viewing of the works and subsequent collective approval to “go public”, a series of site-specific experiential installations of these works and public conversations mediated by the artist and scientist were co-created and presented with the participating families' consent in which the documented engagement of *Lived Lives* families with the artworks became part of the artworks and with bereavement support present. This “ *Lived Lives*” public engagement model has been utilized in both urban and rural community manifestations over the past decade. (see McGuinness & Malone, EPUB, 2019).

***Lived Lives Paper II*.** Here, in *Lived Lives* Paper II, we describe bringing *Lived Lives* to St. Patrick’s University Hospital (SPUH), which is Ireland’s largest and oldest psychiatric hospital. It was founded in 1746 through a donation from Jonathan Swift, Dean of St Patrick’s Cathedral and author of Gulliver’s’ Travels. In its early years it was known as “Swift’s Asylum”. It is now a modern 302-bedded mental healthcare facility in South inner city Dublin with national and international expertize in the multi-disciplinary treatment of moderate to severe mental illness and with a range of in-patient and out-patient programmes (See St Patrick’s University Hospital website) 

## Methods and context

### Building relationships and trust (Affective labour)

The methodology for working with a major psychiatric institution in this study (Bringing *Lived Lives to Swift’s Asylum: A Psychiatric Hospital Perspective*) drew on the design from the public installation of the *Lived Lives* artworks in Letterkenny and in Dunree ( *Lived Lives*, Paper I Cleary *et al.*, 2021) and with The Travellers in Pavee Point ( *Lived Lives: A Pavee Perspective* Malone *et al.*, 2017). It was the destination of a Wellcome Trust-funded project titled “ *Lived Lives: from Tory Island to Swift’s Asylum”,* in which *Lived Lives* manifested in a number of communities between Tory Island and St Patrick’s University Hospital ( McGuinness & Malone, 2019 EPUB). Collaborative pre-planning with Senior SPUH administrators and psychiatrists began in Spring, 2017. A forum was established, and representatives from hospital leadership invited the *Lived Lives* Team into the hospital. The *Lived Lives* model was presented and discussed in detail and the rationale and pathway towards materializing *Lived Lives* with the psychiatric institution Board was outlined and agreed, with its attendant focus on establishing trust, and addressing stigma reduction. As the institution was a busy acute clinical / working site, it was agreed from the outset that the elements of the exhibition would not be located in any of the in-patient units, but could be situated in allied “non-clinical” but adjacent sites, where it would be tended such that patients would not be able to “wander in” to the exhibition (as per community installation protocol set down by *Lived Lives* families). The Board was afforded an opportunity to be heard, on their own terms and expressing their own fears of the Project (the subject matter). The *Lived Lives* model has always included counselling support as part of the project protocol. At least one bereavement counsellor was in attendance during exhibition opening hours, as per the original ethics approval, and per our own protocol that no one would leave any worse than when they entered. The presence of this counselling individual was brought to the attention of participants at the outset of each mediated tour, indicating that support was available. This type of “safety net” has provided reassurance to hosting institutions, and has freed up the research team to focus exclusively on the presentation as it is being delivered (presence), rather than having to double as counsellors also.

The *Lived Lives* project team travelled to the hospital for all follow-up planning meetings. It was agreed that there would be a show-back of the project engagement for hospital personnel prior to public dissemination. Written approval was obtained from St Patrick’s University Hospital Ethics Committee for this study and as per protocol, informed verbal / oral consent (to preserve anonymity) was obtained from all participants prior to taking part (SPUH Protocol 22/17). [“Consent will be obtained verbally by the researcher, which will take place after each Lived Lives tour... Questionnaires will be left to one side for participants to take if they wish to take part (rather than handing them out as this may lead to undue pressure on individuals to take part”)] As per this ethically approved protocol, participants were *not* videoed during the completion of the DEQ Questionnaire or Project Feedback Form, to preserve anonymity. Written ethics approval was also provided by the SVUH Ethics and Medical Research Committee for an amendment to the original *Lived Lives* protocol (SVUH 20/02/2006) to include *Lived Lives* manifesting and being documented in SPUH, and for the completion of the anonymous exhibition feedback form and anonymous Discrete Emotions Questionnaire (SVUH Protocol ID 07/07/2017). As limited literacy has been a feature of some of *Lived Lives* previous iterations, it was advised and considered that verbal / oral informed consent could address informed consent with sensitivity, so as not to create any embarrassment or upset. Before entering the guided exhibition, participants were gathered together in a room adjacent to the exhibition, where story boards were erected describing the history of the project. In this room, the artist and scientist told / informed all of the prospective participants about the exhibition, and its journey and coming together of art and science for a greater understanding and knowledge related to suicide. They were also told “what to expect”, in-that suicide is a sensitive and sad subject, and that the objective of the exhibition was to learn and understand more about suicide through the guided exhibition and the voluntary participation and voluntary feedback of participants, and that no one should leave the guided exhibition “any worse than when they entered”. Participants were invited to ask any questions they had in relation to the exhibition, and they were informed that there was no onus on them to proceed to participation, or any onus to disclose whether or not they were patients at SPUH. Hard copies of “Suicide in Ireland 2003–2008” ( Malone, 2013) (publication) were available for participants to view as an example of output(s) from the project. Participants were also informed that the event would be documented for the purpose of education, training, research and new knowledge and understanding around suicide. Participants were given the opportunity to advise a member of the research team if they did not wish to be documented (audio / visual), and bereavement counsellors in attendance were also introduced. Participants were also informed that the final part of the *Lived Lives* exhibition would involve a reflective conversation among the artworks (in the Hospital Conference Room) which would also be documented (audio / visual) and participants could choose whether or not they wished to participate in this conversation. As with previous public engagements over the past decade, participants were informed that any recording and transcript would be securely stored on encrypted, password-protected hard-drives on the hospital server, backed up daily, and would only be used for educational, training and research purposes. They were also informed these would never be used for commercial purposes. Following the event, a de-identified transcript of the Conference Room conversation was generated for the purpose of this paper, in which any personal identifiers (such as name), or quasi-identifiers that indirectly pointed to the individual’s identity were deleted. Following this verbal / oral consent procedure, consented participants were led into the *Lived Lives* exhibition. When the guided tour was completed (at the Conference Room site), the audience were then invited to take a seat around the table and to provide their insights, feelings and reflections, and to observe whether anyone had regrets about joining the Exhibition. As per ethics protocol, no onus was placed on participants to join or contribute to the conversation. Participants were informed that the documented outputs (audio / visual and transcript) would be used for research, education and understanding about suicide. Attention was drawn to available counselling support. Participants were also invited to complete the anonymous written feedback forms as is usual with *Lived Lives*. This round table Conference Room conversation is transcribed and de-identified in line with ethics protocols described above, and is reported in Results section (see *Underlying data* ( Lived Lives, 2021)). Consent was obtained verbally to protect anonymity and reading assistance was offered to participants if requested. Participants who chose to complete the feedback forms were invited to place their completed questionnaires in the locked and secure *Lived Lives* “ballot box” to further protect anonymity. Participants were not videoed during the completion of the DEQ and Project Feedback forms (as per ethics protocol), and members of the research team were on hand to answer any questions relating to the feedback process. Participants were given at least 15 minute’s rest following the guided exhibition before they completed the Feedback Form and the DEQ. These completed forms were then transferred from the “ballot box” to a locked cabinet in a locked research office in SVUH, where the quantitative and qualitative data were up-loaded into an encrypted password-protected drive on the SVUH server for analyses, which was fire-wall protected and backed up daily.

### Attendees, participants, delegates and installations

Participants at *Lived Lives SPUH* included 58 delegates from the Annual SPUH Founders Day Conference (November 2017), which included mental health professionals, service users, policy makers and senior administrators, and was advertised on the hospital website and was open to the public. Participants (SPUH Founder Day Conference delegates) experienced the mediated LL Exhibition which is summarized as follows:

The Clinical Director at SPUH (JVL) welcomed and introduced the audience to *Lived Lives* team in SPUH Lecture Theatre; Lived Lives team then introduced the project, and invite potential participants to move to a room adjacent to the commencement of the guided exhibition, where all elements of participation are outlined to the group to obtain informed consent (oral) as outlined above.

*21Grams in Group Therapy Room: Making Stigma Visible*;

*Lived Lives - Garden Walk*

*Archive Rooms in the Medical Library*

*Lost Portraits Gallery in the Hospital Conference Room*.

**The process of the mediated journey of *Lived Lives installations at SPUH*** **(**see SPUH mediated tour, Mc Guinness & Malone, 2019 EPUB, page 64)

Following the introduction to the *Lived Lives* team at the Founders Day Conference, SMcG and KMM proposed to the audience that rather than listen to another lecture, delegates were invited to rise from their seats and move through the hospital towards the room adjacent to the Lived Lives Exhibition where informed oral / verbal consent was obtained (as described above). Following this procedure, participants were invited to move into *Lived Lives* as contributors rather than consumers. This audience then moved with the team towards the 1 ^st^ art installation – namely *21 g at SPUH – Making Stigma Visible*.

***21 g at SPUH – Making Stigma Visible***: The artwork had been installed in the Group Therapy Room ( Figure 1). In this iteration, *21 g* consisted of 151 shirt fragments, which was chosen to impart a special mental health-related significance to depression and its treatment. As mentioned above, there are an estimated 302 suicide deaths in Ireland annually that had suffered from depression. This number was halved by the artist to 151, and intended to depict “half the story”. This turn of phrase is often used satirically when a fact / statistic is being under-estimated. Eight shirt fragments also carried the wagon-wheel cultural logo for Travellers on the collar to reflect traveller suicide deaths annually with depression (“half the story” – the estimate is more like at least 16 per year, with an SMR of 6.6). ( All Ireland Traveller Health Study Research Team, 2012). The artist invited the audience to make a fingerprint mark on any of the shirts by dipping their finger in ink and then onto the shirt fabric as a metaphoric means of “making stigma visible” ( Figure 2 a & b).

***Garden Walk:*** Following this engagement, the audience were then led through the hospital garden to avoid any in-patient exposure, and towards the very old part of the hospital building. Unintentionally, the 10 minute walk took on an element of reverence, with a funereal likeness in the failing light, with in-audible quiet conversations emerging from the group in relation to the event along the way.

***Archive Rooms in Medical Library:*** Once the old part of the hospital building was reached, the audience were moved into the Medical Library, where *Archive Rooms* was installed ( Figure 4). Belongings of the deceased were carefully placed by the artist amongst medical and psychiatric textbooks and Journals. Two video screens in the room played footage of *Lived Lives* families engaging with the artworks including *Archive Rooms*. The audience were invited to engage physically with this installation and took time to do so ( Figure 3).

***Lost Portrait Gallery in The Conference Room***: When this ended, the audience were moved next door to The Conference Room where The *Lost Portrait Gallery* was installed around the walls of the room ( Figure 4: Participants engaging with *The Lost Portraits Gallery* installed in the Conference Room, St. Patrick’s University Hospital). A video screen played footage of *Lived Lives* families engaging with the *Lost Portrait Gallery*. It also included a powerpoint slide of written feedback from one of the *Lived Lives* mothers as follows ( *The most profound part of this Visual Arts Autopsy for me was when I went into the circular white room…..I stood in front of her (Fiona’s portrait) and I put my hands either side of her face. There was nobody in the world but us… … I kissed her and walked out of the circular room and stood outside looking in at her for what seemed like an age… …Three days later, I still feel healing and warmth. I felt the best I have felt in 5 years since my beloved 1st born child Fiona (aged 20) handed her life back to God. My block of ice in my chest is thawing”* (Fiona’s mother, Galway, 2009 EPUB Page 2–4,). The Conference Room engagement was the final step on the mediated tour, and participants were invited to consent to join the team at the Conference Room table for conversation and feedback as outlined above.

### Participant feedback

***Questionnaire data from Lived Lives at St. Patrick’s Mental Health Services, Nov 2017*.** Data was collected as part of the *Lived Lives* event which took place in St. Patrick’s University Hospital from the 27 ^th^ to the 30 ^th^ of November (2017) and thus participants in this study were recruited from people who chose to attend the *Lived Lives* mediated exhibition over 3 days. Those who attended included health professionals, mental health service users and members of the general public.

An ethically approved anonymous semi-structure questionnaire was based on feedback forms used at previous *Lived Lives* installations, and consisted of eight questions (see *Extended data* ( Lived Lives, 2021)). There were also spaces provided to record demographic details, patient status and the completion date. The first three questions were directly related to the participants’ thoughts on *Lived Lives* and the next three questions were related to their own personal experience of suicide bereavement and mental health history (including suicidal feelings). As has always been the case, the *Lived Lives* Feedback Form was presented on pink paper, as a satirical nod to the paperwork for involuntary detention in a psychiatric hospital in days past, which was known as “The Pink Form”.

Descriptive statistics were conducted using SPSS Version 25 to assess demographic information about participants. Frequency analyses were then conducted to assess the frequency of the answers “yes”, “no” and “I don’t know” in response to the quantitative questions (questions 1–6) across the sample.

Question 7 was an adapted version of The Discrete Emotions Questionnaire ( Harmon-Jones *et al.*., 2016) which intends to measure self-reported discrete emotions associated with emotional states. The scale measures 32 discrete emotions categorised into 8 broader sub-scales (4 negative and 4 positive): anger, fear, sadness, disgust, anxiety, desire, relaxation and happiness. Individuals were asked “while viewing *Lived Lives*, to what extent did you experience these emotions?” and then instructed to rate each emotion from 1 (not at all) to 7 (an extreme amount). One-way repeated measures ANOVAs were conducted using SPSS to asses which emotions on the Discrete Emotions Questionnaire these individuals felt most strongly after viewing *Lived Lives*. Separate ANOVAS were conducted for the positive subscales (desire, relaxation, happiness) and negative subscales (fear, anger, sadness, disgust, anxiety). 

Finally, question 8 was a qualitative question asking participants about their overall response to the *Lived Lives* works and the option to add any “further comments” relating to the previous questions if they so wished. A brief thematic analysis was conducted on this open-ended question based on methods described by Braun & Clark (2006).

### Evaluation

In addition to written participant feedback, two independent evaluators (Dr. Consilia Walsh - psychiatric clinician and Dr. Aine Phillips - artist) sat in and observed *Lived Lives* “in action” at SPUH and provided written feedback, reflections and assessment. Both evaluators (Dr. Consilia Walsh and Dr. Aine Phillips) provided written informed consent to publishing these evaluations.

## Results

Results I reports on the written feedback (quantitative and qualitative) provided by 68 attendees over the 3 day course of the *Lived Lives* event; Results II reports on the taped *Lived Lives* Conversation in the Conference Room; Results III reports on the external evaluation of *Lived Lives* in SPUH.

### Results I: Participant questionnaire feedback

A total of 68 people completed the questionnaire, ranging in age from 20–73, with a mean age of 42. The sample was 71% female ( *n*= 48). Four participants identified as patients, 53 identified as “not a patient” and 11 participants did not answer this question. 17 of the participants took part in the final day of the *Lived Lives* mediated exhibition as part of the Founders Day Mental Health Conference hosted by the hospital.

Participants’ responses to questions 1–6 about their engagement with Lived Lives are presented in Table 1. 

**Table 1.** *Lived Lives* at SPUH - feedback form responses.

*Q1. Do you think Lived Lives could benefit people* *following a suicide death?*		
Answer	Frequency (n)	%
Yes	62	91
No	-	-
Don’t know	5	7.5
Missing	1	1.5
*Q2. Do you think Lived Lives could somehow * *benefit people with mental health difficulties?*		
Answer	Frequency (n)	%
Yes	60	88
No	1	1.5
Don’t know	6	9
Missing	1	1.5
*Q3. Do you think Lived Lives “glamorizes” and/or* * “romanticizes” suicide?*		
Answer	Frequency (n)	%
Yes	-	-
No	68	100
Don’t know	-	-
Missing	-	-
*Q4. Have you lost anyone to suicide?*		
Answer	Frequency (n)	%
Yes	42	62
No	25	37
Missing	1	1
*Q5. Have you personally experienced any mental* * health difficulties?*		
Answer	Frequency (n)	%
Yes	31	46
No	36	53
Missing	1	1
*Q6. Have you ever experienced suicidal feelings?*		
Answer	Frequency (n)	%
Yes	24	35
No	43	63
Missing	1	2
*If yes, do you think the Lived Lives project/* * exhibition could reduce suicidal feelings?*		
Answer	Frequency (n)	%
Yes	31	46
No	5	7
Don’t know	1	1

### Q7. The Discrete Emotions Questionnaire

In total, 60 participants completed the DEQ in full ( Harmon-Jones *et al.*., 2016). No significant effect on participants’ positive emotions was found after viewing *Lived Lives*, Wilks Lambda =0.98, *F* (2, 58) = 0.64, *p* = >0.05. There was however a significant effect on negative emotions, Wilks Lambda = 0.16, *F* (4, 56) = 70.25, *p* = < 0.05, multivariate partial eta squared = 0.834. The means and standard deviations of these emotions are presented in Table 2 below.

**Table 2.** Discrete emotions questionnaire responses.

Negative Subscales (items)	Mean	SD	*N*
Anger (anger, rage, mad, pissed off)	1.77	1.14	60
Disgust (grossed out, nausea, sickened, revulsion)	1.56	0.87	60
Fear (terror, scared, panic, fear)	1.96	1.25	60
Anxiety (dread, anxiety, nervous, worry)	2.64	1.20	60
Sadness (sad, lonely, grief, empty)	4.12	0.94	60

The questionnaire also gave participants the opportunity to provide qualitative feedback as part of Q.8 which read:

***“Having viewed and experienced Lived Lives what is your response (your immediate thoughts and feelings etc.)? Do you have any further comments on any of the previous questions?”*.**

**Sad and Glad**: Many participants expressed that although they found the exhibition sad and emotional, they were glad they had attended as it had put the issue of suicide in perspective for them and deepened their understanding of the topic e.g.:

*P17 (Female, 25): “Very powerful exhibition, emotional experience. Appreciated all the details that went into this and the thought for the families felt strongly connected with this subject more than ever before”.*

*P51 (Female, 64): “Very moving and emotional. It brought the people who died by suicide to a living state and their families feelings, so real. Thanks for this experience.”*

*P51 (Female, 64): “Very moving and emotional. It brought the people who died by suicide to a living state and their families feelings, so real. Thanks for this experience.”*

**Beneficial:** Mental health professionals who engaged with the *Lived Lives* project also reported finding the experience beneficial e.g.:

*P4 (Female 25): “Excellent exhibition. A very emotional experience, beneficial for everybody to see. Particularly relevant for healthcare professionals in psychiatry as promotes greater understanding of the effects of suicide on victims and their families. Feel I have greater empathy for victims and families”*

*P53 (Female, 53): “Professionally I feel reenergised and refocused on the work needed to do to prevent suicide and reduce the stigma that families and loved ones experience. It is left me with a heightened of responsibility. Finally as an individual it has reinforced the need for compassion for those who struggle within my personal social network and beyond. Thank you so much for your gift of this experience “Lived Lives”. Perhaps it will come back to for another time “enriched post New York” and post APA!”*

*P61 (Female, 41): “A very valuable piece of work for staff working in mental health, for patients (+ ex-patients), for family coping with grief.”*

**Young people should see**: Many participants felt that they exhibition should be seen by many more people, in particular young people e.g.:

*P13 (Male, 26): “It’s an exhibition all young students should be opened to in secondary schools”.*

*P21: (Female, 40): “I think everyone should see this. I wish for all teenagers to see this + talk about suicide + what it means. I think it is really valuable work + I’m glad to have been a part of that, even though it was painful at times + difficult”*

**Thanks to the *Lived Lives* research families**: The qualitative space was also used by many participants to express their gratitude – for their own lives and to the *Lived Lives* families for sharing their stories and to the organisers of the exhibition for providing the opportunity for them to engage with the Lived Lives project e.g.:

*P54 (Female, 51): “Gratitude to the artist, & the families. They have been so brave and courageous. Sadness for all the lives lost and the loved ones left. Pain for the friends lost and their families. Also, because I’ve been there myself but so gracious to be still here”*

*P63 (Female, 44): “Just a profound sense of humanity you have brought to the lives represented in the exhibition – and to the lives of people who commit suicide and their families. And gratitude. Overwhelming gratitude at my life and loves.”*

*P35 (Female, 32): “I am grateful for the organisers, feel this exhibition needs to be viewed by a lot more people, it is so interesting maybe some answers beyond this project and feel a lot is to be said about the lives behind suicide #Mindblowing Thank you”*

Bereavement support is always provided, and is on-hand throughout the event. The research team offered company to those who seemed to be visiting alone. As it happened, the majority of participants had at least one other participant with them. In reality, this support was more of offering companionship, “a quiet word” and personal chaperoning if this offer was taken up. A debriefing session with our Art Therapist for younger participants seemed to be popular and effective (see McGuinness & Malone, 2019, Closings Sessions. EPUB Page 55).

**Results II: *Lived Lives* Conference Room Conversation and participant verbal feedback amongst Lost Portrait Gallery (N=17)** ( Mc Guinness & Malone, 2019, SPUH Conference Room Conversation, EPUB, page 65) (see also *Underlying data* ( Lived Lives, 2021)).

17 attendees (12 females, 5 males) from the Mental Health Conference participated in the *Lived Lives* Conference Room Conversation. They included service users, mental health professionals and senior hospital administrators.

**Immediate feelings**: While discussing their immediate feelings following engagement with *Lived Lives*, many participants agreed they would describe it as “powerful”. M1, a member of the general public, said “its powerful, that’s all I can say. It makes you want to make a difference when you leave this room”. Another participant, a psychiatric trainee in the hospital (F1), described the conflict she has experienced between grieving for those lost by suicide and remaining professional. She described the exhibition as “profoundly human” and described it as “really powerful to be allowed feel”. F1 also highlighted the significance of the *Lost Portrait Gallery* being installed in the Hospital Conference Room “where lots of important decisions are made”, saying “I imagine it’d have been quite stark for the people who had meetings in here this week.” F4 and F5, another two employees at the hospital, also remarked on the “contrast” of the project being in a psychiatric hospital. F4 described the experience as leading her to reflect on her work and she described how speaking about the issue as part of the exhibition had made her “a bit more hopeful”. Similarly, F5, An Administration Director at the hospital, reflected on an earlier conversation about professional failure in terms of suicide deaths saying “out of a sense of failure there is a power in the vulnerability. It can be very empowering to experience something like this and re-invigorate our passion to make a difference”.

For M2, a service user, the *Lost Portrait Gallery* was powerful in a different way: “there is something quite powerful about the fact that we are here – in this room, and statistically I could be on the wall and I’m not, and I’m very grateful I’m not and that gives me encouragement and hope”. F3, another service user, echoed this sense of gratitude saying, “in my own past I have attempted suicide, and I feel a profound sense of gratitude actually at being here that I’m not up on the wall”.

**Any regrets about participating?**: When asked if they had any regrets about attending the event, none were expressed by the participants. F1, psychiatric trainee, expressed no regrets and said “I think I have to say I am even struck by how brave people are about sharing their own personal experiences even in this room, and I think it’s kind of amazing in terms of the power of this exhibition that people feel – open enough and they feel there is a safe enough space to talk about their own experiences. It reminds me of why I became a doctor”. F4, a senior employee in the hospital, said that despite reservations about “crying in front of everyone” before she came to the exhibition, she had no regrets after participating “with these young people in front of you (tapestries) who lost their lives in this way, you wouldn’t be human if you didn’t cry. I don’t mind who sees me cry”. F5, another senior employee at the hospital echoed that she had no regrets in participating and she discussed the considerations the organisation had to make in advance of collaborating with the *Lived Lives* project: “We actually had to give it considerable consideration, because obviously you can see here today, the vulnerability, the humanity that we’ve all experienced, professional or untrained you know, its opened up something and we had to take that very seriously, but I am so so glad we did”. One participant (P26), whilst calling the project “incredible research”, raised an objection to advance notice not being provided regarding video-documenting, and to the nature of how consent to participate was obtained. Her view was acknowledged by the team who reiterated the consent procedure and the verbal notice on documenting the event before the commencement of the mediated tour (and the participant subsequently remained to the end of the mediated tour). Referring to the invitation as part of *Making Stigma Visible* to mark his thumb in ink and leave a mark on one of the 21 grams white shirts and “make stigma visible”, M3, an addictions counsellor, said “I feel privileged to be part of it you know. I don’t know if I want to wash this off or not you know. You know its’ powerful”.

### Siting of *Lived Lives* artworks in and within the psychiatric hospital

The proposal to bring *Lived Lives* into SPUH challenged the institution to confront stigma. The fact the project had backing from the Wellcome Trust, and consistent with their reputation as advanced leaders in mental healthcare (and respectful of the core values of Swift’s vision), and following careful consideration, the Hospital Board elected to take on the project, with a small element of trepidation at the outset, but which dissipated by the time the project was completed, with one of the hospital directors ultimately commenting “I’m so so glad we did”. There was enormous symbolism, meaning and irony in the placement of the artworks within the psychiatric hospital, which was thought-provoking for the participants, and heightened the impact of the *Lived Lives* message of confronting stigma. Some participants chose to reflect on the *Archive Room* being placed within the Medical Library as a way to raise the question about suicide and depression “What do we really know”, and how the *Archive Room* contents presented a deep and different knowledge. One participant was prompted to speak of the challenges associated with divestment in the aftermath of a suicide loss of a loved one, and what one does with their belongings as they are so emotionally charged, observing that the *Lived Lives* experience and especially *Archive Rooms*, helped her to “overcome this”, and see things in a different way.

Others found the placement of the *Lost Portrait Gallery* surrounding the Conference Room walls as particularly poignant, gently but persuasively questioning the authorities who might have attended the Conference Room on hospital business during the week of *Lived Lives* about the problem of suicide deaths. Others found the installation of *21g* in the Group Therapy Room as particularly haunting “If I was to come to this exhibition again, I’d go back to the shirts every time”. Many but not all welcomed the invitation to place a mark “stain” on the shirt fragments as a metaphor for making stigma “visible”. Indeed, one participant shared that he wasn’t sure if he wished to wipe the ink from his thumb, it being a reminder of the profound feeling he had experienced by making a mark. *The Garden Walk* also created a quiet space in the fading light for reflecting as participants moved from *21g Making Stigma Visible* to the remainder of the exhibition in the older part of the hospital.

### Results III: Clinician and artist independent evaluation (Dr. Consilia Walsh, Dr. Aine Phillips)

A priori, both evaluators elected to assume an ethnographic approach to the *Lived Lives* experience - observing, engaging and reflecting through writing. To this end, both drew on their wealth of professional experience in their respected fields of clinical science and art. The brief summary of their evaluations provided here foregrounds their most salient points (see full texts in *Underlying data* ( Lived Lives, 2021)). Objective feedback from the senior clinician psychiatrist (CW) drew attention to “the journey” aspect of the event, beginning with the anonymous shirt collars of *21g*, moving on into *Archive Room*, where you get to know the deceased a little better, and then into *The Lost Portrait Gallery*, where “identity” is revealed. CW also articulated how *Lived Lives* communicates the sensitivity around suicide and suicide bereavement in a very different and tangible way. As a clinician, she has been dealing with suicide and attempted suicide all her clinical life, and found the Lived Lives project touched and moved her in a new and compelling way. She also reflected on how this form of communication could resonate so well with young people, encouraging them to speak out as they did in the videos. Artist AP chose to reflect on aesthetics and meaning. For example, she sees *21g* as a “throng of anthropomorphic collars which gives comfort in its evocation of unity”, suggesting that the collars are “spectral traces of the lost individual lives”, representing succour or solace in the subtle beauty and tactile substance of the artwork”. “Viewers”, she says, are encouraged to touch the collars, “with ink stained thumbs to leave their own printed trace upon the installation”. This act of touching she observes denotes “physical contact which in turn enables a psychological touching to simultaneously happen, allowing the participant to intellectually and emotionally grasp this complex subject”. AP suggests that *Lived Lives* “gives audiences access to shared authorship in the work and the means to make a difference in the way the subject of suicide is mediated and disseminated. McGuinness and Malone have enabled the participating families to define and direct how their loved one’s stories are depicted in the project so this relational approach was maintained throughout”. AP observes that the project explores and reveals layers of invisible knowledge - private thoughts and feelings of suicide-bereaved individuals are given public disclosure within a supported, safe and imaginative context. This she says allows for the dissolving of the private and public in therapeutic ways to help dismantle stigma. She says that *Lived Lives* is a collective experience, often in silence and contemplation alongside dialogue and interaction and this provides for a necessary and effective processing of the subject to take place. AP concludes that the *Lived Lives* authors invite us to align our deeper selves with the artworks and, via the art, we are helped to address suicide as a meaningful life experience. She says that the work does not “deny our tragedies”, but becomes “a legitimate place for solemn emotions and creates a dignified home for both sorrow and hope” (see *Underlying data* for complete CW and AP evaluations ( Lived Lives, 2021); for other evaluations to date see McGuinness & Malone, 2019, EPUB Page 68–96).

A show-back of “ *Lived Lives*, A psychiatric hospital perspective” was arranged for six weeks following the event in SPUH, with notably favourable feedback from attendees, and with no objections expressed towards pursuing its dissemination for education, training and research.

## Discussion

Transposing a dynamic, mediated, interactive and collaborative science-arts project about suicide into a psychiatric institution was a ground-breaking experience, which to our knowledge has not previously been attempted. Our findings identified a deep stigma that still surrounds mental illness and everything associated with it. In this study and in their own words, those who serve to support and treat all kinds of mental illness, witness and experience stigma in their daily work. When the stigma of suicidal thoughts and fatal deeds is added to the stigma of mental illness and the stigma of having been in a psychiatric hospital, it seems to create a pervasive muting of voice and a response of recoil and intervention paralysis. Situated within the *Lived Lives* project lies a catalyst for reconciliation and change, where an aperture for voice is created through a transformative process attendant in the artworks and facilitated through the mediated safe-space experience. *Lived Lives* sits comfortably in discomfort, unafraid to call out the home-truths about stigma and its pervasive and pernicious impact, managing to do so with deepest respect and empowerment, and with restoring identity at its core.

Our previous *Lived Lives* iteration in Dunree, Co. Donegal demonstrated that *Lived Lives* could work well with people with previous suicidal experiences. Cleary *et al.*, 2021 ( *Lived Lives* Paper I). Many of the participants in the *Lived Lives* SPUH project acknowledged their own suicidal feelings, previous suicidal feelings and acts, and the experience of suicide bereavements, with an affirmative and sharing disclosure to the Conference Room conversation.

No participant thought the *Lived Lives* experience glamorized, glorified, romanticized or fantasized suicide, with many stating the opposite, as it focusses on the harsh reality for the deceased and the bereaved. As one of the *Lived Lives* families bluntly put it, “dead is dead” (Suzanne – Galway, 2009. see EPUB Page 2–4).

The DEQ was included to assess emotional responses following the engagement *with Lived Lives*. Consistent with our expectation, the participants reported nothing positive about the emotional response, but instead experienced significant negative emotional feelings consistent with a “visceral response” frequently reported by participants. Many participants describe the experience as “powerful”. In a previous iteration of *Lived Lives* with young rural men, one of the participants described its impact as “being like you were punched in the stomach”, clasping his belly as he spoke (EPUB Page 71). The value of the DEQ requires further research ( Harmon-Jones *et al.*., 2016)

The *Lived Lives* project contains elements of contemporary anti-stigma / stigma-reduction strategies ( Knaak *et al.*, 2014; Knaak *et al.*, 2017) such as Ecological Validity, where our target audiences are working partners from the outset and are set in the richness of their natural environment. The *Lived Lives* families are co-curators with Lived Experience from the outset. Human Design contains Human Factors, Empathic Design and Design Thinking, all of which are represented in the conceptualization, construction, realization, manifestation and delivery of *Lived Lives*. Human Centeredness and Empathy informs the *Lived Lives* process and is essential for understanding the experiences of patients and their families as they relate to stigma, as these represent the core start and end points of any anti-stigma effort or program. Collaboration, Curiosity, Optimism and Experimentation have kept the project fresh, relevant, relatable and nimble, and evaluation is integral to the process. *Lived Lives* has been described as a community intervention (see Christabel Owens evaluation and Figure, in Malone *et al.*, 2017), and could be considered “an intervention prototype” ( Ungar *et al.*, 2016).

There are limitations with regard to aspects of the study. Advertising for such an event is challenging. The event was posted on the hospital website where it was stated that it was open to the public. Perhaps the fears of visiting an event hosted in a psychiatric institution were a stigma-related deterrent. One way or another there are barriers to reaching the public and attracting them to participate in such projects around suicide and stigma ( Knaak *et al.*, 2017). Previous iterations of *Lived Lives* in rural communities have also had a mixed public response, where media interviews with scientist and artist on local radio stations have proven to be effective ( McGuinness & Malone, 2019), EPUB page 99).

In addition to the evaluations by an independent scientist and artist in this paper, multi-source and multi-disciplinary evaluation of the *Lived Lives* Project has been provided by six further diverse sources of expertize over the past decade, embracing science arts and humanities, bearing witness and sharing their testament and testimony ( McGuinness & Malone, 2019; EPUB, page 68–96). Their guiding and generous insights provide a deep, grounding and enduring critique, with an inherent value to the project being greater than the sum of their parts. As such, all these evaluations need no introduction, translation or interpretation, and speak for themselves as they enrich the project. In this vein, both evaluators (Walsh and Phillips) provided particular insights, reflection and understanding (see *Underlying data* ( Lived Lives, 2021)).

The triple stigma of mental illness, suicidal experiences and having been in a psychiatric hospital has never been quantified. It is unknown if there is an additive, cumulative or multiplier effect of each. Clinically, these stigmas impact on self-esteem and impede recovery, which warrants further research ( Moses, 2014; Rüsch *et al.*, 2005). Projects such as *Lived Lives* have the potential to meet stigma head-on, and can operate within the context of suicide intervention and prevention planning (as evidenced with *Lived Lives* in Donegal (EPUB Pages 59–63). *Lived Lives* has existed in the public domain since 2010. Over 2,000 members of the public have engaged and interacted directly with the Project, with at least 20% of those being young adults. Also included are 1 ^st^ responders such as police and coastguard, LGBT, Irish Travellers, Health policy makers, health providers and service users. Many others have interacted with the project through *Lived Lives* Lectures and Conversations, such as over 2,000 University College Dublin medical students – (the “doctors of tomorrow”). No adverse events have been reported to date, whereas an overwhelming majority of participants have shared their positive testimony either orally, or written or both, and many have returned for repeat engagement with the project. *Lived Lives* in this instance was experienced in a psychiatric hospital as authentic at a deep level of humanity. The challenge is to move it forward for greater exposure in communities nationally and internationally, and further into psychoeducation and anti-stigma whilst preserving its essence.

## Data availability

### Underlying data

Irish Social Science Data Archive: Lived Lives, https://www.ucd.ie/issda/data/livedlives/ ( Lived Lives, 2021). Study number (SN): 0070-00 

This project contains the following underlying data:

- Lived_Lives_Quantitative_Data_Paper2_SPUH (Excel file containing quantitative data collected at Lived Lives SPUH)
- Lived_Lives_Conference_Room_Transcript_Paper2_SPUH (Word file containing anonymised transcription of post-exhibition interactive conversation with the Lived Lives team)
- Lived_Lives_Paper2_SPUH_Qualitative_Feedback (Word file containing quantitative data collected at Lived Lives SPUH)
- Lived_Lives_Walsh_and_Phillips_Feedback_and Evaluation (Word document containing evaluations of Lived Lives by Dr. Consilia Walsh and Dr Áine Phillips)

These data are under restricted access due to the sensitivity of the subject material. To access the data, please complete a ISSDA Data Request Form for Research Purposes, sign it, and send it to ISSDA by email ( issda@ucd.ie). Researchers will be asked to provide a description for the intended use of the data and will be asked to agree to the terms of use, as outlined in the request form. Data access will be granted for teaching and research purposes under ISSDA terms and conditions.

### Extended data

Irish Social Science Data Archive: Lived Lives, https://www.ucd.ie/issda/data/livedlives/ ( Lived Lives, 2021). Study number (SN): 0070-00 

This project contains the following underlying data:

- Lived Lives SPUH Blank Questionnaire (Copy of questionnaire used at Lived Lives SPUH)

Data are available under the terms of the Creative Commons Attribution 4.0 International license (CC-BY 4.0).
