# Supplementary material for: Bringing Lived Lives to Swift’s Asylum: a psychiatric hospital perspective
Source: Wellcome Open Res. 2022 Mar 29;6:85. Originally published 2021 Apr 19. [Version 3] doi: 10.12688/wellcomeopenres.15588.3 (PMC8980674; doi:10.12688/wellcomeopenres.15588.3)
Supplement: Peer review history

# Peer review: Reviewer response for version 3

**DOI:** 10.21956/wellcomeopenres.19725.r49557

**Published:** 2022-04-04

**Authors:** Rahul Shidhaye

**Affiliations:** 1 Pravara Institute of Medical Sciences, Pune, Maharashtra, India

I had a chance to look at the revised manuscript and I am satisfied with all the responses.

Is the work clearly and accurately presented and does it cite the current literature?

Yes

If applicable, is the statistical analysis and its interpretation appropriate?

Yes

Are all the source data underlying the results available to ensure full reproducibility?

Yes

Is the study design appropriate and is the work technically sound?

Yes

Are the conclusions drawn adequately supported by the results?

Yes

Are sufficient details of methods and analysis provided to allow replication by others?

Yes

Reviewer Expertise:

Global Mental Health, Yoga and Health, Implementation Science

I confirm that I have read this submission and believe that I have an appropriate level of expertise to confirm that it is of an acceptable scientific standard.

# Peer review: Reviewer response for version 2

**DOI:** 10.21956/wellcomeopenres.19095.r47950

**Published:** 2022-03-09

**Authors:** Rahul Shidhaye

**Affiliations:** 1 Pravara Institute of Medical Sciences, Pune, Maharashtra, India

Malone and colleagues present mixed-methods evaluation of 'Lived Lives' project in this extremely well-written paper. I read this paper sitting in my office in a small village in rural Maharashtra (western part of India), yet I felt that I was part of the various exhibits in St. Patrick's University Hospital. I agree with the feedback from the participants that this was very emotional, moving, and extremely powerful. I indeed felt sad, but glad and as I was reading the paper, I started reflecting on what I could do as a psychiatrist to address the issues related to suicide, bereavement, and stigma. I reviewed the revised version of the paper. Dr. Quinlivan had made some excellent comments on the methodology and other aspects of the paper. Authors have revised the paper based on these suggestions and Dr. Quinlivan seems to be satisfied with these changes. As a result, I do not have any major comments on this version except for a few which follow: 

1. It will be good to have clearly stated objectives and rationale for this particular paper described at the end of the introduction section. On page 6, authors have included a paragraph about 'Lived Lives II' and in this same paragraph, some of these details can be included. 
2. Similarly, an overview of the evaluation plan can be presented in the methods section before going into the details of each of the methods. Importantly, explain why a particular method was chosen and how it relates to other methods in this study. 
3. Making stigma visible is a stated objective of this paper. It will be good if you can present any data (qualitative) related to this theme. I found most of the things related to this as some kind of statements from authors, but not entirely supported by the qualitative data. Maybe I am missing something here. 
4. Mean and standard deviation for each of the sub-scales of Discrete Choice Questionnaire is presented. For lay readers (like me), it will be good to explain these scores in context. 
5. I agree that 'Lived Lives' is a potential community intervention to address stigma, suicide, and bereavement issues. But the results presented in this paper only suggest that the exhibit and guided tour is acceptable to those who attended. What was the effect of this on their internalized/externalized stigma, emotional state, and long-term behavior is not presented here. Additionally, it is also important to explore ways to engage with wider community. It will be good if authors can elaborate on this in the discussion section, particularly highlighting the limitations of the current evaluation plan. 

Is the work clearly and accurately presented and does it cite the current literature?

Yes

If applicable, is the statistical analysis and its interpretation appropriate?

Yes

Are all the source data underlying the results available to ensure full reproducibility?

Yes

Is the study design appropriate and is the work technically sound?

Yes

Are the conclusions drawn adequately supported by the results?

Yes

Are sufficient details of methods and analysis provided to allow replication by others?

Yes

Reviewer Expertise:

Global Mental Health, Yoga and Health, Implementation Science

I confirm that I have read this submission and believe that I have an appropriate level of expertise to confirm that it is of an acceptable scientific standard, however I have significant reservations, as outlined above.

# Response

**Published:** 2022-03-24

**Authors:** kevin malone

**Affiliations:** ucd, Ireland

Dear Dr. Shidhaye,

 Thank you for taking the time to review this paper and for your generous feedback.

 Based on your comments, we have made the following changes:

 We have added a clear objective at the end of the introduction section to orientate the reader. We have also added an overview of the evaluation plan earlier in the methods section as suggested and elaborated on the nature of the methodology for this and why it was chosen. We have also added in explanations for the terms "mean" and "standard deviation" in the results section and elaborated on the limitations of the current research in terms of its cross-sectional nature with regard to emotional state. We have further reflected on the limitations with regards to engaging the wider community who chose not to attend the exhibition and how we might find out more about their choice in this regard.

 In response to your comments on the theme of "making stigma visible" we would like to clarify that this was an overall theme for the project in SPUH and for the changes made to the 21g artwork. Thus, it refers to a metaphor for what we hoped would be achieved through installing the exhibition in this space as opposed to a research objective to be measured or a "theme" in the qualitative data sense. We hope that answers your query in this regard.

 We would like to take this opportunity to thank you again for your constructive comments. Your input and indeed the input of all reviewers have helped us improve the quality and structure of our paper as we continue to navigate the best ways to present this research which doesn't conform to the traditional "intro, methods, results, discussion" format.

 Kind Regards,

*Lived Lives *Research Team

# Peer review: Reviewer response for version 2

**DOI:** 10.21956/wellcomeopenres.19095.r47711

**Published:** 2021-12-21

**Authors:** Leah Quinlivan

**Affiliations:** 1 NIHR Greater Manchester Patient Safety Translational Research Centre, The University of Manchester, Manchester Academic Health Science Centre, Manchester, UK; 2 Centre for Mental Health and Safety, University of Manchester, Manchester, UK

All queries are well addressed in the revised version. No further changes are requested. 

 This is excellent paper, well written, and emotive to read. To truly transform mental health services at a human level and address societal stigma, we need these innovative and groundbreaking ‘Lived Lives’ collaborations between art and science. I couldn't sum up the impact of this work greater than the participants in the event:

 “there is something quite powerful about the fact that we are here – in this room, and statistically I could be on the wall and I’m not, and I’m very grateful I’m not and that gives me encouragement and hope”” (M2, person with lived experience)

 “out of a sense of failure there is a power in the vulnerability. It can be very empowering to experience something like this and re-invigorate our passion to make a difference”. (F5, Administration Director)

*“Very moving and emotional. It brought the people who died by suicide to a living state and their families feelings, so real. Thanks for this experience.” (P51)*

 Best of luck to the team in their innovative endeavours. I look forward to seeing this intervention develop its transformative reach further into society.

Is the work clearly and accurately presented and does it cite the current literature?

Yes

If applicable, is the statistical analysis and its interpretation appropriate?

Yes

Are all the source data underlying the results available to ensure full reproducibility?

Yes

Is the study design appropriate and is the work technically sound?

Yes

Are the conclusions drawn adequately supported by the results?

Yes

Are sufficient details of methods and analysis provided to allow replication by others?

Partly

Reviewer Expertise:

Public engagement, sensitive research methods, suicide prevention, self-harm, clinical research.

I confirm that I have read this submission and believe that I have an appropriate level of expertise to confirm that it is of an acceptable scientific standard.

# Peer review: Reviewer response for version 1

**DOI:** 10.21956/wellcomeopenres.17070.r44154

**Published:** 2021-06-23

**Authors:** Leah Quinlivan

**Affiliations:** 1 NIHR Greater Manchester Patient Safety Translational Research Centre, The University of Manchester, Manchester Academic Health Science Centre, Manchester, UK; 2 Centre for Mental Health and Safety, University of Manchester, Manchester, UK

This novel study evaluates the use of a psychobiographical and visual arts autopsy led engagement event as an ‘intervention’ to address the stigma of suicide in the community. This combination of art and science brings forward the identity of the person lost to suicide into the public domain. This unique approach to public engagement involves researchers, clinicians, artists, families, and communities to facilitate dialogue of highly stigmatised and sensitive topics, in a safe but challenging space. Based on evidence from previous engagement events, the authors situated the mediated exhibition within a psychiatric institution, where experiences of stigma are persistently high.

 Hosting an event such as this in health services is of great significance. Despite progress in the development of mental health services, patients frequently speak of receiving stigmatising comments when they seek help after harming themselves (Quinlivan et al., 2021 a,b). Statistics surrounding suicide can also create distance between professionals and the devastating human experience when someone loses their life to suicide.  Novel engagement approaches such as these, are needed to bring forward the lived life of those lost to suicide compassionately, whilst challenging the stigma surrounding suicidal behaviour.

 The authors conducted a mixed-methods evaluation of the exhibition which was attended by 86 participants. Emotional responses were intense but well managed with openly available bereavement support. Most participants felt the engagement event could be helpful after suicide and could help reduce suicidal feelings.

 This is a ground-breaking and important programme of work which leads the way internationally for co-production and public engagement in suicide prevention and mental health research. The authors have taken highly stigmatised and often abstract concepts and placed them in the reality of the devastating loss when someone dies by suicide. The publication is strong and interesting/ emotive to read. Some minor comments which the authors may which to address prior to indexing.

  

1. In the first section on depression and suicide in Ireland. The authors use the term ‘post-attempter’. Can this be replaced by an alternative term such as ‘post suicide attempt’, to be consistent with the ethos of the paper.
2. Some of the images are a blurry. Suspect this is due to uploading issues. Please do check these prior to submitting a new version. The photographs are outstanding and an integral part of the publication.
3. The term ‘show-back’ is used in various places throughout in the document. May be helpful to define or re-word this term. I have no idea what it means.
4. Some restructure and tightening of the methods may be helpful to readers.
5. In the abstract [method section]. May be helpful to be clearer about the methods. Something like ‘a mixed method approach was used to evaluate an arts-led intervention to address stigma’.
6. The method section may benefit from a slight restructure, and additional use of headings (e.g., ethical approval/ ethics approach, procedure(s), questionnaire, participants, analyses).
7. It would be helpful to see a more detailed analyses section. Why did the authors use ANOVA? What is a ‘brief’ thematic analyses?
8. There are a few different numbers for participants in the text. A defined participant section may help make this clearer. For example, in the abstract it states 86 people engaged with the exhibition, but in the attendees there are 58 delegates, and later in the results 68 people completed the questionnaire. Appreciate there is different levels of engagement and completion of the questionnaire, but can this be made clearer for the readers?

Overall, this is an excellent paper. I fully agree with the authors of the work:  

*“Lived Lives *sits comfortably in discomfort, unafraid to call out the home-truths about stigma and its pervasive and pernicious impact, managing to do so with deepest respect and empowerment, and with restoring identity at its core.”

 References:

 Quinlivan, L., Gorman, L., Littlewood, D. L., Monaghan, E., Barlow, S. J., Campbell, S., ... & Kapur, N. (2021). ‘Wasn’t offered one, too poorly to ask for one’–Reasons why some patients do not receive a psychosocial assessment following self-harm: Qualitative patient and carer survey. *Australian & New Zealand Journal of Psychiatry*, 00048674211011262. https://doi.org/10.1177/00048674211011262

 Quinlivan, L. M., Gorman, L., Littlewood, D. L., Monaghan, E., Barlow, S. J., Campbell, S. M., ... & Kapur, N. (2021). ‘Relieved to be seen’—patient and carer experiences of psychosocial assessment in the emergency department following self-harm: qualitative analysis of 102 free-text survey responses. *BMJ open*, *11*(5), e044434. http://dx.doi.org/10.1136/bmjopen-2020-044434

Is the work clearly and accurately presented and does it cite the current literature?

Yes

If applicable, is the statistical analysis and its interpretation appropriate?

Yes

Are all the source data underlying the results available to ensure full reproducibility?

Yes

Is the study design appropriate and is the work technically sound?

Yes

Are the conclusions drawn adequately supported by the results?

Yes

Are sufficient details of methods and analysis provided to allow replication by others?

Partly

Reviewer Expertise:

Public engagement, sensitive research methods, suicide prevention, self-harm.

I confirm that I have read this submission and believe that I have an appropriate level of expertise to confirm that it is of an acceptable scientific standard, however I have significant reservations, as outlined above.

## References

[1] : 'Wasn't offered one, too poorly to ask for one' - Reasons why some patients do not receive a psychosocial assessment following self-harm: Qualitative patient and carer survey. *Aust N Z J Psychiatry* .2021; 10.1177/00048674211011262 48674211011262 10.1177/00048674211011262 34015945PMC8941717

[2] : ‘Relieved to be seen’—patient and carer experiences of psychosocial assessment in the emergency department following self-harm: qualitative analysis of 102 free-text survey responses. *BMJ Open* .2021;11(5) : 10.1136/bmjopen-2020-044434 10.1136/bmjopen-2020-044434 PMC814943334024759

# Response

**Published:** 2021-12-08

**Authors:** kevin malone

**Affiliations:** ucd, Ireland

Dear Dr Quinlivan

 Thank you for taking the time to review our paper and for your valuable insights and comments.

 Based on your feedback, we have changed the term "post-attempter" to "post suicide attempt" and acknowledge the original language used was an oversight and not in line with the ethos of our paper nor our research.  We have also added a definition of the term "show-back", which we hope will clarify this important part of the research process for readers.

 The novel nature of this arts-science collaboration and its methods can present a challenge in terms of structuring papers in the traditional "introduction, methods, results, discussion, format".  We have however, based on your feedback, attempted to clarify the methodology used in the abstract and also added some additional information specific to analyses used with the questionnaire data collected. Furthermore, we have clarified participant numbers throughout the paper and thank you for bringing this important point to our attention as we can see it may have been confusing for readers to keep track of the different types of "participants" we had.

 We hope you find these changes have improved the quality of our paper and we thank you again for your input.

 Kind Regards,

*Lived Lives *Research Team
